# The Guideline Implementability Decision Excellence Model (GUIDE-M): a mixed methods approach to create an international resource to advance the practice guideline field

**DOI:** 10.1186/s13012-015-0225-1

**Published:** 2015-03-15

**Authors:** Melissa C Brouwers, Julie Makarski, Monika Kastner, Leigh Hayden, Onil Bhattacharyya, the GUIDE-M Research Team

**Affiliations:** McMaster University, Juravinski Campus, G Wing, 2nd Floor, Room 207, 1280 Main Street West, Hamilton, ON L8S 4L8 Canada; McMaster University & the Escarpment Cancer Research Institute, Hamilton, ON Canada; Li Ka Shing Knowledge Institute of St. Michael’s Hospital, Toronto, ON Canada; Women’s College Hospital, Toronto, ON Canada; Women’s College Hospital & University of Toronto, Toronto, Ontario Canada

**Keywords:** Clinical practice guidelines, Practice guidelines, Implementability, Implementation

## Abstract

**Background:**

Practice guideline (PG) implementability refers to PG features that promote their use. While there are tools and resources to promote PG implementability, none are based on an evidence-informed and multidisciplinary perspective. Our objectives were to (i) create a comprehensive and evidence-informed model of PG implementability, (ii) seek support for the model from the international PG community, (iii) map existing implementability tools on to the model, (iv) prioritize areas for further investigation, and (v) describe how the model can be used by PG developers, users, and researchers.

**Methods:**

A mixed methods approach was used. Using our completed realist review of the literature of seven different disciplines as the foundation, an iterative consensus process was used to create the beta version of the model. This was followed by (i) a survey of international stakeholders (guideline developers and users) to gather feedback and to refine the model, (ii) a content analysis comparing the model to existing PG tools, and (iii) a strategy to prioritize areas of the model for further research by members of the research team.

**Results:**

The *Gu*ideline *I*mplementability for *D*ecision *E*xcellence *M*odel (GUIDE-M) is comprised of 3 core tactics, 7 domains, 9 subdomains, 44 attributes, and 40 subattributes and elements. Feedback on the beta version was received from 248 stakeholders from 34 countries. The model was rated as logical, relevant, and appropriate. Seven PG tools were selected and compared to the GUIDE-M: very few tools targeted the *Contextualization and Deliberations* domain. Also, fewer of the tools addressed PG appraisal than PG development and reporting functions. These findings informed the research priorities identified by the team.

**Conclusions:**

The GUIDE-M provides an evidence-informed international and multidisciplinary conceptualization of PG implementability. The model can be used by PG developers to help them create more implementable recommendations, by clinicians and other users to help them be better consumers of PGs, and by the research community to identify priorities for further investigation.

## Background

Evidence-based practice guidelines (PGs) have become internationally accepted knowledge tools to support quality agendas aimed at improving health outcomes and health systems [1,2]. While the potential for PGs is strong [3-5], impacts on processes and outcomes of care are not consistently reported [4,5]. This has led to a demand for more implementable PGs by clinicians, system leaders, and the public in our increasingly result-oriented healthcare environment. Implementability refers to characteristics of the PGs that promote their use [6], and these may be both intrinsic attributes—those related to the PG itself—or extrinsic attributes—those related to the actions of the healthcare system in which the guidelines are used. If minor changes to the intrinsic attributes of the guideline, such as what content is presented and how the content is presented, could achieve change in evidence-based care and outcomes, it might be a very low-cost strategy for a substantial benefit, and so well worth exploring [7].

Support of this notion has led to several advancements in the PG enterprise [8-14]. For example, the Appraisal of Guidelines Research and Evaluation II (AGREE II) is an international PG quality assessment tool that can be used to inform PG development and reporting [8-10]. There are tools (GuideLine Implementability Appraisal [GLIA] [6]), frameworks (Implementability Framework [11]), and coordinated research efforts (e.g., Guideline Implementability Research and Application Network [GIRANet] [12]) to support the implementability of guidelines. Further, PG development standards and checklists have been released that address this issue including those developed by the Institute of Medicine (IOM [2]), the Guidelines International Network (G-I-N [13]), and the Grading of Recommendations Assessment, Development, and Evaluation (GRADE) Group [14]. Despite guidance and resources to support PG development, variability in PG quality and application remains [15]. More effort is required.

We contend that the PG implementability field has not thoroughly explored or integrated learnings from the range of disciplines that study behavior change and communication. Moreover, there is a lack of common nomenclature, labels, and definitions in the implementation field making scientific advancements and building upon what is known slow, fragmented, and, at times, duplicative. In addition, differentiating between PG implementability features supported by evidence and linked to process and clinical outcomes, from those that are not, is often not well articulated.

To address these limitations, we analyzed the literature and engaged the international community to create a resource targeted primarily for PG developers and the PG scientific community that provides a more comprehensive approach to the implementability of PGs. In this program of research, our objectives were as follows:

1. To create a comprehensive and evidence-informed model of guideline implementability:
  Provide common (English-language) nomenclature (labels, definitions) appropriate across geographic jurisdictions and disciplines to facilitate communication and logical progression of scientific inquiry.
  Seek support for the model from the international PG community through a formalized external consultation.
2. To identify priority areas of further investigation:
  Examine the extent to which major international PG development, reporting, and evaluation tools address the components of the final model.
  Identify those components in the model that are perceived to be more and less well studied.

This paper reports on how our model, the *GU*ideline *I*mplementability for *D*ecision *E*xcellence *M*odel (GUIDE-M), was created and how it can be used by PG developers and other knowledge users to optimize the implementability of PGs and by researchers to advance the science and application of PGs.

## Methods

Ethics approval for the study was granted from the Hamilton Integrated Research Ethics Board (McMaster University and Hamilton Health Sciences, Hamilton, Canada; #09-398). This project was funded by the Canadian Institutes of Health Research (CIHR): design and implementation of the study protocol, analysis and interpretation of the data, and the writing of this paper were independent of the funders.

### Development of the model

#### Foundational work: developing the evidence base using realist review

To develop the evidence base, a realist review was used to identify intrinsic features of PGs related to their implementability [7,16]; these data were to serve as the basis of the model. Realist review encourages the interrogation of theories and mechanisms underlying an area and consideration of diverse literatures [7]. Three search strategies (i.e., a systematic search, a targeted search, and a reference list search) of articles in the medical, human factors, psychology, management/marketing/business, information technology, sociology, and graphic design fields were conducted. For each relevant article, the following data elements pertaining to the intrinsic PG features were extracted: discipline of article, feature label, definition, and operationalization as defined by the authors. A series of iterative steps were used to synthesize the raw data features into like groupings (synonyms, antonyms, similar linguistic roots), clustering and combining the features into logical thematic groupings using the definitions provided by the primary source, and crafting a common label and operational definition for each of the core features and groupings. The detailed methods and results of the realist review have been published separately [7,16] and will only be summarized in this paper.

#### Creation of a conceptual model

Using the data from the realist review, the next step was to organize and create logical relationships between the core features of implementability. Through an iterative process, a series of consensus meetings with the core research team were used to structure the data to create global domains and to categorize features across and within different conceptual domains, reflecting the relationships among them. An overall operational definition was crafted for each of the global domains. Seven formal meetings were undertaken over the course of 18 months to create the beta version of the GUIDE-M.

### External consultation

#### Participants

We recruited participants from various relevant PG communities including international PG development groups, members of the G-I-N, and knowledge translation and implementation science researchers. Our objective was to be as inclusive as possible and to enable a voice for those interested in being part of the process; a specific sample size was not sought.

#### Process

Participants who agreed to participate received an e-mail with their unique identifier number and the URL to the on-line survey platform supported by Limesurvey™. E-mail invitations and a series of two reminders were sent to candidate participants. The survey ran from June to August and October to November of 2013. Participants were introduced to the purpose of the project and half were randomized to review an overview video introducing the model first^a^. Using a formal survey with seven-point Likert response scales, participants were asked for feedback on (i) the structure of the model and how concepts were categorized and organized, (ii) labels used to identify its components, and (iii) operational definitions. This feedback was sought for the overall model and, specifically, for two perspectives of the model: (i) the upper two layers of the model comprised of key tactics and the domains that underpin these tactics and (ii) the lower layers of the model comprised of the domains and the subdomains and lower order components that underpin these domains. All participants were provided with a visual aid of the relevant section and perspective of the model using Mindmeister™, an on-line collaborative mapping program.

#### Analysis

Descriptive analyses (mean [*M*], standard variation [SD], range [*R*], frequency [*F*]) for each item on the survey were calculated. *A priori*, the team determined items that would be prioritized for modification in cases where agreement (ratings >5) was not achieved by 80% of respondents or the median score was <5. Written feedback was reviewed and analyzed by themes across the GUIDE-M components. Modifications to any components of the model were conducted through consensus among members of the core research team resulting in the final model.

### Content analysis: GUIDE-M coverage by existing PG tools and resources

Many tools have been designed to improve the quality of the PG enterprise by providing direction on how best to develop, report, and/or evaluate PGs. We sought to examine the extent to which these tools addressed any of the GUIDE-M components and, where coverage did exist, for what function (development, reporting, and/or evaluation). To this end, seven international PG tools were selected, and their contents were compared to the core components of the GUIDE-M. The selection criteria used to guide the choice of tools were as follows: availability in the public domain; emerged as part of our realist review^b^; provided practical advice related to development, reporting, or appraisal of some aspect of PGs; and were perceived by our research team as having traction and high profile within the international PG community. The tools were the AGREE II [8], IOM standards [2], G-I-N standards [13], Guidelines 2.0 [14], ADAPTE [17], GLIA [6], and GRADE [18]. In contrast to the others, the GLIA [6] tool is described specifically in the context of guideline implementability.

For each core component of the GUIDE-M, two members of the team independently coded whether each of the tools provided adequate information or advice on how the component should be developed (D) and/or reported (R) in a guideline and/or informed how the component should be appraised (A). Disagreements were resolved by consensus. The coded output had final confirmation by the research team.

### Expert opinion of research priorities

Members of the research team were asked to independently consider the final GUIDE-M and its content relative to existing PG tools to provide their expert opinion on whether additional research efforts directed toward (i) development tools, (ii) reporting tools, and (iii) appraisal tools were warranted. In making these judgments, they were asked to consider the extent to which PG tools and resources exist, whether these tools and resources were sufficient, and whether additional research/work would yield important gains. For each item, a five-point scale was used (1 = low priority for additional work to 5 = high priority for additional work). Descriptive statistics were generated for each component to determine overall estimate of priority and the consensus of the opinions. An *a priori* decision was made to operationalize lower priority as mean response scores between 1 to 2.5, medium priority as mean response scores >2.5 and <3.5, and higher priority as mean scores >3.5.

## Results

### Development of the beta version of the model

The realist review search strategies yielded 2,550 potentially relevant citations for consideration. From the 367 eligible articles, 1,571 uniquely labeled data elements were originally extracted [16]. Using these data, the beta version of the model was crafted comprising six layers; the terms tactics, domains, subdomains, attributes, subattributes, and elements were used to denote each of the layers as they move toward greater specificity and narrowness. The beta version of the GUIDE-M comprised two core implementability *tactics*, six implementability *domains*, 16 *subdomains*, 27 *attributes*, and 22 (together) *subattributes* and *elements*.

### External consultation

Survey data regarding the beta version of the model were received from 248 individuals. The majority were women (58.1%) and 60.4% were between the ages of 46–65 years. Respondents represented 34 countries: 50.8% from North American countries, 31.0% from European countries, 10.4% Australasian countries, 1.2% South American countries, 4% African countries, and 1.2% did not answer the question.

The feedback was universally positive. On a scale of seven, participants understood the beta version of the GUIDE-M (*M* = 6.2, SD = 1.0) and agreed with it (*M* = 5.8, SD = 1.2). Starting at the higher layers of the GUIDE-M structure, participants agreed that separating the key tactics of *Creation of Content* and *Communication of Content* was a logical way to think about guideline implementability (*M* = 6.1, SD = 1.3), and they agreed the tactic labels were appropriate (*M* = 6.1, SD = 1.1). Within the middle layer of the GUIDE-M structure, and as reported in Table 1, participants agreed that the manner in which the domains were clustered within each of the two tactics was logical and that domain labels were appropriate (*M* range = 5.7 to 6.5). Finally, in the lower layers of the GUIDE-M structure, and as reported in Table 2, participants agreed that the manner in which the subdomains and lower order components (attributes, subattributes, and elements) were clustered within each of their domains was logical (*M* range = 5.8 to 6.2), relevant to guideline implementability (*M* range = 6.0 to 6.3), and appropriately labeled (*M* range = 5.7 to 6.1). Overall scores for the appropriateness of the subdomains were positive (*M* range = 5.8 to 6.2). Extensive written feedback was also provided aimed to strengthen and make more explicit the model, the labels it used, and the descriptions provided.

**Table 1:** **Participants’ ratings (mean and standard deviations (SD)) of (i) logic of domain clusters underpinning Creation of Content (Content) and the Communication of Content (Communication) tactics and (ii) appropriateness of domain label names in the beta version of GUIDE-M**

**Component of beta version of GUIDE-M**			**Mean**	**SD**
Content tactic	*Logic of structure*		6.0	1.0
	*Appropriateness of domain names*	*Domain names*		
		Stakeholder involvement	6.3	1.0
		Evidence synthesis	6.5	0.8
		Considered judgment	5.7	1.4
		Feasibility	5.9	1.3
Communication tactic	*Logic of structure*		6.4	0.9
	*Appropriateness of domain names*	*Domain names*		
		Message	6.1	1.3
		Format	6.3	1.1

Maximum rating: 7; minimum rating: 1.

**Table 2:** **Participants’ ratings (mean (** ***M*** **) and standard deviations (SD)) of GUIDE-M structure and nomenclature: logic of subdomain clusters within each domain [logic], relevance of subdomains to higher order domain [relevance], appropriateness of subdomain labels [appropriateness], and appropriateness of subdomains to the overall beta version of GUIDE-M**

**Ratings**	**Domains of content tactic**								**Domains of communication tactic**			
	**Stakeholder involvement** ^**a**^		**Evidence synthesis** ^**b**^		**Considered judgement** ^**c**^		**Feasibility** ^**d**^		**Message** ^**e**^		**Format** ^**f**^	
	***M***	**SD**	***M***	**SD**	***M***	**SD**	***M***	**SD**	***M***	**SD**	***M***	**SD**
Logic	6.2	1.1	6.1	1.1	6.1	1.0	6.3	1.0	6.4	0.9	6.1	1.1
Relevance	6.3	1.1	6.0	1.2	6.1	0.9	6.2	0.9	6.3	0.9	6.1	1.0
Appropriateness	6.0	1.1	5.7	1.4	5.9	1.1	6.0	1.1	6.1	1.1	5.9	1.2
Overall	6.1	1.2	5.8	1.3	5.9	1.2	6.2	0.9	6.3	0.9	6.0	1.2

Maximum rating: 7; minimum rating: 1.

^a^Subdomains of stakeholder involvement.

^b^Subdomains of evidence synthesis.

^c^Subdomains of considered judgment.

^d^Subdomains of feasibility.

^e^Subdomains of message.

^f^Subdomains of format.

### Final GUIDE-M, GUIDE-M coverage by existing PG tools, and future research priorities

#### Final GUIDE-M

Using the quantitative survey and descriptive feedback from the external consultation, an iterative consensus process with the research team was conducted to refine the model. The final GUIDE-M is comprised of six levels starting with (i) 3 core tactics, (ii) 7 domains, (iii) 19 subdomains, (iv) 44 attributes, and (v) 40 (together) subattributes and elements. The full model is provided on Table 3. As a means to manage the complex data set, our description will focus on the top three layers (tactic, domain, and subdomain). We refer readers to other publications reporting the results of the realist review [16] and the on-line version of GUIDE-M (guide-m.ca) for more detail on the lower order components (see the “Discussion” section below).

**Table 3:** **Final GUIDE-M**

**Tactic**	**Domain**	**Subdomain**	**Attribute/subattribute/element** ^**a**^
Developers of content	Comprehensive	Clinical experts	Multidisciplinary and multijurisdictional
			Researchers and users
		Target population	Individual patients
			Family members
			Groups representing patients
		Decision makers	Multidisciplinary and multijurisdictional
			Researchers and users
		Methodologists	Practice guideline experts
			Knowledge synthesis experts
			Health economics experts
			Ethicists
			Implementation experts
	Knowledgeable and credible	-	-
	Competing interests	Financial	-
		Professional and/or academic	
		Advocacy	
Creating content	Evidence synthesis	How: execution of methods to develop evidence base	Systematic and reproducible
			Valid and reliable
		What: completeness of reporting evidence base	Question
			Eligibility criteria
			Literature search strategy
			Critical appraisal
			Data extraction
			Data synthesis
			Reporting
		When: currency of evidence base	-
	Deliberations and contextualization	Clinical applicability	Clinical relevance
			Patient relevance
			Implementation relevance
		Values	Patient/client
			• Acceptability
			• Preferences
			Provider
			• Acceptability
			• Preferences
			• Clinical flexibility
			• Clinical judgment
			Guideline developer
			• Acceptability
			• Preferences
			Population/societal
			• Acceptability
			• Preferences
			• Diversity
			• Equity
			Policy
			• Acceptability
			• Preferences
		Feasibility	Local applicability
			• Local adaption
			• Application tools and strategies
			Resources
			• Availability of resources
			• Economic evaluation
			Novelty
			• Compatibility
			• Knowledge and skills
Communicating content	Language	Simple	Succinct
			Uncomplicated
		Clear	Actionable
			• Specific
			• Unambiguous
			Effective writing
		Persuasive	Framing
			Relative advantage
	Format	Version	Tailored
			Modalities
			• Electronic (dynamic, static)
			• Non-electronic
			Document types
		Components	-
		Presentation	Document layout
			• Visual elements
			• Length
			Structure
			• Match system to the real world
			• Grouping/ordering
			Information visualization
			• Display (tables, algorithms, pictures, graphical display)
			• Context (framing, vividness, depth of field, evaluability)

^a^Subattribute (bulleted), element (parentheses).

At its highest level, the three core tactics aimed to improve the implementability of PGs serve as the foundation of the GUIDE-M: (i) *Developers of Content*, (ii) *Creating Content*, and (iii) *Communicating Content*. The *Developers of Content* tactic is comprised of three domains: *comprehensive*, *knowledgeable and credible*, and *competing interests*. This tactic advises on the types and characteristics of participants who ought to be recruited to create a comprehensive multidisciplinary PG development group, the expected skills of the group members, and issues related to competing interests of the group members. Optimized stakeholder involvement and participation strategies will increase credibility and acceptability of resulting recommendations. The *Developers of Content* is a new tactic emerging from restructured components in the beta version of the model and requests from the external reviewers to add this as an explicit section.

The *Creating Content* tactic is comprised of two core domains: *evidence synthesis* and *deliberations and contextualization*. The *evidence synthesis* domain outlines how to create the evidence base, how to report it, and how to ensure its currency. The *deliberations and contextualization* domain refers to the process of moving from the evidence to recommendations through the careful consideration of the clinical applicability, values of PG stakeholders (patients, providers, policy makers, society, and developers), and issues of feasibility in applying the recommendations.

The *Communicating Content* tactic includes two domains, *language* and *format*. This tactic focuses on specific strategies to communicate PG information to optimize its implementability. This includes how to create clear, simple, and persuasive messages and how to format messages into key components while also considering presentation styles and the design of multiple versions to address the needs of different users.

#### GUIDE-M coverage by existing PG tools

Table 4 reports the overlap between the GUIDE-M components and the content and functions (development, reporting, and evaluation) of seven PG tools. As seen, the number of tools to support operationalization of the GUIDE-M varies across its components and varies as a function of the tools (i.e., development, reporting, and evaluation). For example, the AGREE II was assessed to provide (i) good development, reporting, and appraisal advice for most of the subdomains reflected in *comprehensive*, *competing interests*, and *evidence synthesis* domains and (ii) good development, reporting and appraisal advice to about half of the subdomains within the *deliberations and contextualization*, *language*, and *format* domains. In contrast, Guidelines 2.0 and ADAPTE were assessed to provide good development advice (but incomplete or no reporting or appraisal advice) for most of the subdomains in the GUIDE-M.

**Table 4:** **GUIDE-M coverage by existing guideline tools**

**GUIDE-M**				**Guideline tools**						
**Tactic**	**Domain**	**Subdomain/attribute**		**AGREE II**	**IOM Stds**	**G-I-N Stds**	**Guidelines 2.0**	**ADAPTE**	**GLIA**	**GRADE**
Developers of content	Comprehensive	Clinical experts		D, R, A	R	D	D	D	-	-
		Target population		D, R, A	D, R	D	D	D	-	-
		Decision makers		D, R, A	D	D	D	D	-	-
		Methodologists		D, R, A	D, R	D	D	D	-	-
	Knowledgeable and credible			-	-	-	-	-	D, A	-
	Competing interest	Financial		D, R, A	D, R	R	D	D	-	-
		Professional or academic		D, R, A	D, R	R	D	D	-	-
		Advocacy		D, R, A	D, R	R	D	D	-	-
Creating content	Evidence synthesis	How: execution of methods to develop evidence base		D, R, A	D	R	D, R	D	D, A	D
		What: completeness of reporting evidence base		D, R, A	D, R	R	D	D	-	D
		When: currency of evidence base		D, R, A	D, R	R	D	D	-	D
	Deliberations and contextualization	Clinical applicability	Clinical relevance	D, R, A	D	R	D	D	D, A	D
			Patient relevance	D, R, A	D	-	D	D	D, A	D
			Implementability relevance	D, R, A	D	-	D	D	D, A	D
		Values	Patients/clients	D, R, A	D	-	D	D		-
			Provider	D, R, A	D	-	D	D	D, A	-
			Guideline developers	-	D	-	D	D		-
			Population/societal	-	D	-	D	D		-
			Policy	-	-	-	D	D	-	D
		Feasibility	Local applicability	D, R, A	D	-	D	D	D, A	-
			Resources	D, R, A	D	-	D	D		D
			Novelty	-	D	-	-	D	D, A	-
Communicating content	Language	Simple		D, R, A		-	D	-	D, A	-
		Clear		D, R, A	D, R	R	D	D	D, A	-
		Persuasive		-	-	-	-	-		-
	Format	Versions		-	-	-	D	-	D, A	-
		Components		D, R, A	-	R	-	D	D, A	-
		Presentation		-	-	-	D	-	D, A	-

*D* development, *R* reporting, *A* appraisal.

Overall, across the GUIDE-M, the greatest number of tools cover the *evidence synthesis* domain of the model and likely reflect considerable duplication of effort. The *language* domain, the *format* domain, and some components of the *values and feasibility* subdomains have the fewest number of tools. One subdomain of the *language* domain, *persuasiveness*, which is extensively discussed in the management literature but not in the medical literature, is not covered by any tool. Finally, there are far fewer tools with a function that targets the appraisal of content than there are tools that target development issues; reporting tools fall in the middle.

### Expert opinions of priorities

Table 5 summarizes averaged ratings provided by five members of the research team who prioritized each component of the GUIDE-M. The *comprehensive*, *evidence synthesis*, and *format* domains were rated as lower priority by the respondents in contrast to the *deliberations and contextualization* domain and the *language* domain which were rated as medium and higher priority for additional research work. In addition, continued work in the appraisal function was viewed as a greater research priority than additional efforts in the development and reporting functions.

**Table 5:** **Expert opinion of research priorities as a function of GUIDE-M component and priority area**

**GUIDE-M**				**Priority area**		
**Tactic**	**Domain**	**Subdomain/attribute**		**Development**	**Reporting**	**Appraisal**
Developers of content	Comprehensive	Clinical experts		L	L	L
		Target population		L	L	L
		Decision makers		M	L	M
		Methodologists		L	L	L
	Knowledgeable and credible			M	M	H
	Competing interest	Financial		L	M	M
		Professional or academic		M	M	M
		Advocacy		M	H	H
Creating content	Evidence synthesis	How: execution of methods to develop evidence base		L	L	M
		What: completeness of reporting evidence base		L	L	M
		When: currency of evidence base		L	L	H
	Deliberations and contextualization	Clinical applicability	Clinical relevance	M	M	H
			Patient relevance	M	M	H
			Implementability relevance	M	M	H
		Values	Patients/clients	H	H	M
			Provider	H	H	H
			Guideline developers	M	H	H
			Population/societal	H	H	H
			Policy	H	H	H
		Feasibility	Local applicability	M	M	H
			Resources	M	M	H
			Novelty	M	M	M
Communicating content	Language	Simple		M	M	H
		Clear		M	H	H
		Persuasive		H	M	H
	Format	Versions		L	L	L
		Components		L	L	M
		Presentation		L	L	M

*H* high-research priority, *M* medium-research priority, *L* low-research priority.

## Discussion

### Key findings and advancing the field

In this paper, we provide a comprehensive, current, and multidisciplinary model on practice guideline implementability that describes key factors associated with the uptake of PG recommendations. The GUIDE-M comprises three key tactics, seven domains, and multiple subdomains, attributes, subattributes, and elements. Its structure and the operational definitions of each of its components are perceived as logical, appropriate, and relevant by a large sample of members of the international guideline community. The scores were high on all evaluation metrics. The model was used to assess which dimensions are covered by existing PG tools and to identify gaps and duplication in the field.

The GUIDE-M adds to the existing literature by elucidating a more comprehensive model of the implementability of recommendations. First, it identifies where existing international PG-related tools fit and where gaps exist. For example, many tools exist to support the components reflected in the *Developers of Content* tactic. These include the AGREE II [8], the IOM Standards [2], the G-I-N standards [13], ADAPTE [17], and Guidelines 2.0 [14]. Though not the object of the study, it is also acknowledged that procedural manuals published by individual PG development groups also target these areas [19,20]. Similar arguments can be made for most aspects of the *evidence synthesis* domain from the *Creating Content* tactic. In these areas, additional work is likely not required.

In contrast, a key research gap in this area is the *currency of guidelines* attribute. While there is common recognition of the importance of the concept and there are reasonable candidate methods [21], how best to achieve a rigorous yet efficient and feasible way to update PGs requires further investigation. Similarly, while there is consensus about the importance of *deliberations and contextualization* in creating recommendations, less understood are the best strategies to operationalize, report, and evaluate these processes. For example, there is a dearth of methods and tools, informed by evidence, on how best to gather the values of PG developers and populations and how to report these values or appraise the process. The AGREE Enterprise [22] and the GRADE group [23] have programs of research aimed to address such gaps. Finally, the *Communicating Content* tactic is associated with few tools that provide explicit and usable direction to optimize the development, reporting, or appraisal of the *format* and *language* domains. GLIA has led the way [6], and the GRADE/DECIDE group has made significant advances in creating new platforms to present PG recommendations (e.g., GRADE-Pro [24]).

### Strengths and limitations of the study

Strengths of this study are that the GUIDE-M was designed by an international and multidisciplinary team of PG developers, users, and researchers and used rigorous methods. It included meaningful engagement of the larger PG stakeholder community through its iterative evaluation of the beta version of the model. It is grounded in the literature from a broad range of disciplines, beyond the traditional focus on a single discipline. In this way, we believe the GUIDE-M will make an important contribution to advancing the practice and science of PGs implementability in an efficient and accelerated fashion.

There are some limitations of this study. First, and as described, 248 individuals provided feedback to the beta version of the model during the external consultation. This represents a portion of all possible international PG users and researchers, and the potential of a self-selection bias exists. However, the absolute number of respondents and the range of jurisdictions from which they came help mitigate some of this risk. Second, due to resource constraints, the final GUIDE-M was not vetted by a group independent of the research team. Third, the model does not provide data on the relative importance of each of its components. Based on our review of the literature, we do not have sufficient evaluative evidence to argue that certain aspects of implementability should take precedence over another. Moreover, it is likely that the impact and value of components may vary as a function of outcomes (e.g., user satisfaction versus practice change); these data are currently being analyzed. Finally, the expert opinion of our team assessed the priorities for research. While beyond the scope of this project to get confirmation of these priorities by external stakeholders, we acknowledge other experts may support different priorities.

## Conclusion

There are many PG development groups. For example, the National Guideline Clearinghouse has entries representing over 350 clinician and professional authoring groups [25]. With so many players involved, having a common and accessible model that will help foster the creation of high-quality, unbiased, and usable recommendations, and that can facilitate better communication among members of the PG community, is desirable. The GUIDE-M provides a comprehensive analysis of implementability features and a common nomenclature that are fundamental to these goals. It also provides details of the extent to which some existing PG tools and methods support these aims and where there are gaps.

The GUIDE-M will be instrumental to the guideline enterprise. For the PG developer community, the GUIDE-M identifies and operationalizes a suite of features from the lens of implementability: from the composition and phenotype of developers, to what information should be communicated, to how the information should be communicated. Using this knowledge in combination with existing complementary PG tools, developers will be more apt to create usable and useful guidance. For the PG research community, GUIDE-M provides a common structure and nomenclature to improve communication about, and a common understanding of, the implementability literature. Moreover, it provides direction by identifying where there are knowledge gaps, based on expert opinion, and by contrasting that to where there has been sufficient advancement (or duplication of effort) and where additional efforts may only yield marginal improvements. Indeed, even among the seven tools we considered in this study, there appears to be overlap in their scope, intent, and purpose; at times their differences are poorly articulated, small, or nuanced. This risks confusing the PG developer, user, and the research communities. Thus, we believe the GUIDE-M can be used to help structure the direction of scientific inquiry, provide a dialog between the developer and research communities, and improve the quality of inquiry so as to accelerate creation of new needed methods that will ultimately be useful to developers, help avoid future duplication of effort, and create robust and consistent improvements in PG acceptability and recommendation uptake.

To contribute to the PG research enterprise, our team has created an on-line platform, the GUIDE-M Website [26], a resource that is designed to provide users with access to our data and to facilitate development of a common language. It is comprised of three key sections: (i) the complete GUIDE-M structure, (ii) a codebook (the link between each component of the model and information about how it can be used; examples, context, and setting in which it has been studied; evidence of its link to uptake; and reported risks and tradeoffs), and (iii) a bibliography of the references used to create and support the model. Further, to engage the PG community, the resource includes a “wiki” to facilitate community feedback on GUIDE-M, the sharing of knowledge regarding GUIDE-M, and its ongoing refinement by stakeholders outside of the research team.

We hope to use the GUIDE-M and the GUIDE-M Website as a viable platform by which the research and knowledge-user communities can work collectively and quickly to improve guidelines and increase quality of care.

### Endnotes

^a^The video was part of a complementary study aimed to determine if the video assisted in participants’ understanding of the complexity of the GUIDE-M. No differences were found between participants who were and were not randomized to view the video. The video can be found at URL: guide-m.ca.

^b^Guidelines 2.0 [14] was published after the completion of our realist review but was identified by a member of the research team (HS) and was subsequently included.
